# The prevalence of clarithromycin-resistant *Helicobacter pylori* isolates: a systematic review and meta-analysis

**DOI:** 10.7717/peerj.15121

**Published:** 2023-03-30

**Authors:** Mohammad Sholeh, Saeed Khoshnood, Taher Azimi, Jasem Mohamadi, Vahab Hassan Kaviar, Marzieh Hashemian, Somayeh Karamollahi, Nourkhoda Sadeghifard, Hedayat Heidarizadeh, Mohsen Heidary, Morteza Saki

**Affiliations:** 1 Department of Microbiology, Pasteur Institute of Iran, Tehran, Iran; 2 Clinical Microbiology Research Center, Ilam University of Medical Sciences, Ilam, Iran; 3 Student Research Committee, Ilam University of Medical Sciences, Ilam, Iran; 4 Department of Bacteriology and Virology, School of Medicine, Shiraz University of Medical Sciences, Shiraz, Iran; 5 Department of Pediatrics, School of Medicine, Emam Khomeini Hospital, Ilam University of Medical Sciences, Ilam, Iran; 6 Department of Microbiology, School of Medicine, Ilam University of Medical Sciences, Ilam, Iran; 7 Department of Laboratory Sciences, School of Paramedical Sciences, Sabzevar University of Medical Sciences, Sabzevar, Iran; 8 Cellular and Molecular Research Center, Sabzevar University of Medical Sciences, Sabzevar, Iran; 9 Department of Microbiology, Faculty of Medicine, Ahvaz Jundishapur University of Medical Sciences, Ahvaz, Iran

**Keywords:** Clarithromycin, Meta-analysis, Antibiotic resistance, *Helicobacter pylori*

## Abstract

**Background:**

Knowledge of global clarithromycin (CLA)-resistant rates of *Helicobacter pylori* (*H. pylori*) is crucial for decision of the most appropriate eradication therapies with good clinical outcomes. Therefore, this review and meta-analysis aimed to evaluate the global prevalence of the CLA resistance in *H. pylori* to provide some guidance for selecting the first-line antibiotics.

**Method:**

A comprehensive search was performed for relevant literature until April 2021 in PubMed, Embase, and Web of Science databases. Freeman-Tukey double arcsine transformation was performed to estimate the weighted pooled prevalence of resistance.

**Results:**

The meta-analysis included 248 articles. The prevalence of CLA-resistant *H. pylori* was 27.53% (95% CI [25.41–29.69]). The heterogeneity between reports was significant (I^2^ = 97.80%, *P* < 0.01). The resistance rate increased from 24.28% in 2010–2017 to 32.14% in 2018–2021 (*P* < 0.01). Iran, with 38 articles, has the most report. Nevertheless, Switzerland, Portugal, and Israel had the highest resistance rates (67.16%, 48.11%, and 46.12%, respectively). The heterogeneity between the continents and the antimicrobial susceptibility methods also interpreted standard guidelines and breakpoints was insignificant (*P* > 0.05).

**Conclusion:**

Overall CLA resistance rate was 27.53%, worldwide. The difference in CLA resistance rate among the included studies can be due to several reasons such as differences in antibiotic prescription rates in various geographic areas, use of different breakpoints or inaccurate criteria in performed studies, and the emergence of multidrug-resistant (MDR) strains.

## Introduction

*Helicobacter pylori* is one of the most successful human pathogens that affects approximately 50% of the population worldwide. In developing countries 70% to 90% of the population are infected by this bacterium (Arenas et al., 2019; Kocsmár et al., 2021). *H. pylori* infection is related to many gastric diseases, such as peptic ulcers, chronic gastritis, uninvestigated and functional dyspepsia and mucosa-associated lymphoid tissue lymphoma, and even increases the risk of gastric cancer (Savoldi et al., 2018). As for the high prevalence of the bacterium and its related diseases, proper treatment is very important. Today, standard treatment is a three-stage drug that consists of an acid neutralizer and two antibiotics, clarithromycin (CLA), and amoxicillin or metronidazole for 14 days (Hosseini et al., 2021).

However, treatment is difficult because the bacterium quickly develops resistance to the few antibiotics known to be effective (Park et al., 2016). The World Health Organization (WHO) has classified it among the 12 most resistant bacteria in the world (Essaidi et al., 2022). The increasing failure rate of eradication treatment due to the appearance of resistant *H. pylori* strains contributes to the worldwide prevalence of this infection and subsequent inflammatory and neoplastic disorders. Unfortunately, nowadays, the success of this treatment is less than 80% worldwide (Kocsmár et al., 2021; Hussein, Al-Ouqaili & Majeed, 2022).

CLA has been emerged as the basis for *H. pylori* treatment in combined therapy because of small effect on gastric acidity, its low minimal inhibitory concentration, and relatively good mucosal diffusion (Marques et al., 2020; Nishizawa & Suzuki, 2014). Due to extensive usage of CLA in some geographical regions, global prevalence rate of CLA resistance is increasing (Zou et al., 2020). In developing countries, CLA resistance and frequency of re-infection are factors that contribute to high worldwide prevalence of *H. pylori* infection and subsequent inflammatory and neoplastic disorders (Alarcón-Millán et al., 2016). In most European countries, as well as the rest of the world, the prevalence of CLA resistance has reached 20%. With rare exceptions, it is no longer recommended to include CLA in empirical treatment in regions where primary resistance to this antibiotic is 20% (Alarcón-Millán et al., 2016; Morilla et al., 2019).

Knowledge of global CLA-resistant rates of *H. pylori* is crucial for decision of the most appropriate eradication therapies with good clinical outcomes. Therefore, the aim of current review and meta-analysis is to evaluation of the global prevalence of the CLA resistance in *H. pylori*.

## Method

### Search strategy

A comprehensive search was conducted by two researchers in the online databases PubMed, Embase, and Web of Science until April 2021, using relevant keywords such as clarithromycin, antibiotic resistance, and *H. pylori*, as well as related MeSH terms (see Supplemental File 1 for the search syntax). The search syntax is available in Table 1.

**Table 1:** A systematic search including PubMed, Embase, and Web of Science with relevant keywords such as clarithromycin, antibiotic resistance, and *Helicobacter pylori*.

First author (Reference)	Country	Enrollment time	Published year	Type of study	N. patients	Mean age	N. HP	N. Clarithromycin-resistant	AST method	Breakpoint
Horie et al. (2020)	Japan	2005–2018	2020	RET	5,249	58.3	1300	426	MIC	1
Haddadi et al. (2020)	Iran		2020	CS	280	46	128	3	DD	CLSI 2015 21
Eisig et al. (2011)	Brazil		2011	PCS	54	46.6	39	3	MIC	1
Aftab et al. (2016)	Bangladesh	2014–2014	2015	CS	133	35.2	56	22	MIC	0.25
Ortiz et al. (2019)	Honduras	2013–2013	2019	CS	189	54	116	13	MIC	0.5
Silva et al. (2018)	Portugal	2013–2017	2018	PCS	74	14	58	7	MIC	1
Almeida et al. (2014)	Portugal	2009–2013	2014	PCS	180	43.4	180	90	MIC	1
Ilie et al. (2011)	Romania		2011	CS	100	Range: 19–80	70	22	DD	>20 CLSI 2010
Vécsei et al. (2011)	Austria	2007–2009	2011	RET	96	10.8	96	16	MIC	1
Ranjbar & Chehelgerdi (2018)	Iran	2016–2017	2018	CS	700	Range: 3–72	526	335	DD	21
Hamza et al. (2018)	Egypt		2018	CS	150		20	12	DD	21
Gong et al. (2020)	South Korea	2017–2018	2020	RET	13		46	38	MIC	0.5
Wang et al. (2020)	China	2018–2019	2020	CS	124		124	44	MIC	0.5
Su et al. (2022)	Taiwan	2009–2019	2021	RET	87	13.5	65	15	MIC	1
Sugimoto et al. (2017)	Japan	2011–2015	2016	RET	111	55.2	111	90	MIC	1
Abadi et al. (2011)	Iran	2009–2009	2011	CS	210	40.7	197	89	DD	30
Teh et al. (2014)	Malaysia		2014	CS	110		102	7	MIC	1
Peng et al. (2017)	China	2013–2014	2017	CS	178	41.6	78	38	MIC	1
Hashemi et al. (2019)	Iran	2015–2016	2019	CS	150		157	38	MIC	1
Lauener et al. (2019)	Switzerland	2013–2017	2019	CS	140		140	96	MIC	1
Domanovich-Asor et al. (2020)	Israel	2015–2019	2020	CS	48		48	26	MIC	1
Wu et al. (2015)	Taiwan	2010–2014	2015	RET	137		137	95	MIC	1
Vala et al. (2016)	Iran	2011–2012	2016	CS	80		20	4	MIC	0.5
Omar et al. (2014)	Australia		2014	CS	11	46.8	11	8	MIC	1
Vilaichone et al. (2016)	Thailand	2013–2013	2016	CS	291	46.6	124	7	MIC	0.5
Lee et al. (2014)	South Korea	2003–2013	2014	PCS	2,202	52.9	475	147	MIC	1
Lee et al. (2019)	South Korea	2014–2018	2018	PCS	85	55.2	74	24	MIC	1
Goudarzi et al. (2016)	Iran	2014–2014	2016	CS	65	42	65	28	MIC	1
Karpinski et al. (2015)	Poland	1998–1999 2013–2014	2015	CS	108		108	9	MIC	1
Miyata et al. (2021)	Japan	2007–2018	2020	CS	119	12	45	26	MIC	1
Palmitessa et al. (2020)	Italy	2017–2018	2020	CS	224	48.6	92	49	MIC	0.5
Hung et al. (2021)	Taiwan	2016–2019	2021	RET	197	54.8	62	9	MIC	1
Miftahussurur et al. (2016)	Japan	2012–2012	2016	CS	146	42.2	42	9	MIC	0.25
Siddiqui et al. (2016)	Pakistan	2008–2013	2016	CS	889	35.6	92	5	MIC	0.5
Sugimoto et al. (2014)	Japan	2009–2013	2014	CS	153		153	64	MIC	1
Jolaiya et al. (2020)	Nigeria		2020	CS	492		104	41	MIC	0.5
Pandya et al. (2014)	India	2008–2011	2014	CS	125		80	47	DD	30
Lehours, Siffré & Mégraud (2011)	France	2009–2009	2011	CS	127		43	26	MIC	0.5
Sun et al. (2018)	China		2018	CS	49	Range: 27–76	43	9	MIC	0.75
Dekhnich et al. (2018)	Russia	2009–2017	2018	CS	783	51.8	276	16	MIC	0.5
Sugimoto et al. (2020)	Japan	2015–2019	2020	RET	307	62.3	307	102	MIC	1
Siavoshi, Saniee & Malekzadeh (2018)	Iran		2018	CS	450	44.1	104	37	MIC	2
Szadkowski, Zemlak & Muszynski (2018)	Poland	2005–2015	2018	CS	154		55	15	DD	21
Costa, Soares & Goncalves (2017)	Portugal	2012–2016	2017	RET	42	48.9	42	36	DD	17
Aguilera-Correa et al. (2017)	Spain		2016	CS	136		84	48	MIC	0.5
Akar et al. (2021)	Turkey	2018–2019	2021	CS	422	50	133	25	MIC	0.5
Yula et al. (2013)	Turkey	2010–2011	2012	CS	110	41.4	79	7	MIC	1
Zhang et al. (2019)	China	2015–2016	2018	CS	150		149	104	MIC	1
Macin et al. (2015)	Turkey	2006–2012	2015	CS	311	Range: 5–19	93	28	MIC	1
Auttajaroon et al. (2019)	Thailand	2017–2017	2019	CS	93	54.5	70	9	MIC	0.5
Eghbali et al. (2016)	Iran	2012–2013	2016	CS	89	53.6	89	5	MIC	1
Wu et al. (2014)	Taiwan		2014	CS	231		43	5	MIC	1
Kocazeybek et al. (2019)	Turkey	2014–2017	2019	CS	63	47.08	63	24	MIC	1
Egli et al. (2020)	Switzerland	2013–2017	2020	CS	76		76	49	MIC	1
Khani, Talebi Bezmin Abadi & Mohabati Mobarez (2019)	Iran	2017–2018	2019	CS	81	56.8	61	13	MIC	0.5
Morimoto et al. (2015)	Japan		2014	RET	135	62.3	135	35	MIC	1
Alarcón-Millán et al. (2016)	Mexico		2016	CS	144	48.3	45	8	DD	18
Tamayo et al. (2017)	Spain	2013–2015	2017	CS	6,228		1986	349	MIC	1
Yoon et al. (2014)	South Korea	2005–2010	2014	RET	204	52.5	212	18	MIC	1
Miftahussurur et al. (2017)	Dominican		2017	CS	158	47.1	64	2	MIC	8
Mohammad et al. (2011)	Iran	2007–2007	2011	CS	263		84	19	MIC	1
Ha et al. (2019)	Vietnam	2012–2017	2018	CS	185	42.3	104	56	MIC	1
Tanih, Ndip & Ndip (2011)	South Africa		2011	CS	254	44.5	200	40	MIC	1
Yeganeh et al. (2019)	Israel	2016–2016	2019	PCS	218	42	218	96	MIC	1
Liu et al. (2019)	China	2010–2017	2019	RET	1,463		1463	296	MIC	0.5
Zhu et al. (2013)	China	2002–2006	2012	CS	365		365	42	MIC	1
Farzi et al. (2019)	Iran	2014–2015	2018	CS	97	Ranging 10–70	40	14	MIC	0.25
Abdollahi et al. (2019)	Iran	2017–2018	2019	CS	191	38.2	63	20	DD	21
Lee et al. (2019)	South Korea	2015–2018	2018	CS	1,422		140	43	MIC	0.5
De Francesco et al. (2014)	Italy	2011–2012	2014	CS	82		82	42	MIC	0.5
Seo et al. (2013)	South Korea	1990–1994 2005–2009	2013	CS	91	11.8	91	10	MIC	1
Kouitcheu Mabeku et al. (2019)	Cameroon	2013–2015	2019	CS	140		140	19	DD	21
Yin et al. (2020)	China	2016–2016	2016	CS	267	9.4	169	57	MIC	1
Chen et al. (2018)	China		2018	CS	12		12	6	MIC	1
Kakiuchi et al. (2020)	Japan	2018–2018	2020	CS	71	14.7 years	23	7	MIC	0.5
Cuadrado-Lavín et al. (2012)	Spain	2010–2010	2011	CS	76		68	10	MIC	2
Gehlot et al. (2016)	India	2011–2014	2015	CS	68	Range: 18–86	68	8	MIC	0.5
Ogata, Gales & Kawakami (2014)	Brazil	2008–2009	2014	CS	77	11.1	77	16	MIC	1
Eng et al. (2015)	Canada	2012–2013	2015	CS	301		20	8	MIC	0.5
Alarcón et al. (2017)	Spain	2007–2014	2017	CS	824	26	824	422	MIC	0.5
Akhtereeva et al. (2018)	Russia	2011–2013	2018	CS	76	13.6	30	9	DD	30
Selgrad et al. (2013)	Germany	2005–2012	2013	RET	436	51.7	159	12	MIC	1
Gunnarsdottir et al. (2017)	Iceland	2012–2013	2017	PRO	613	57	105	9	MIC	1
Mahmoudi et al. (2017)	Iran	2014–2015	2017	CS	90	9.4	32	7	MIC	1
Shokrzadeh et al. (2011)	Iran	2007–2008	2010	CS	92	45 ± 18 M 38 ± 14 F	42	6	MIC	1
Savari et al. (2010)	Iran	2009–2009	2010	CS	191	Range: 14–84	63	19	DD	21
Shu et al. (2018)	China	2012–2014	2018	CS	1,390	9.5	545	112	MIC	8
Mosites et al. (2018)	USA	2000–2016	2018	CS	763	52	800	238	MIC	1
Parra-Sepúlveda et al. (2019)	Chile	2005–2007 2015–2017	2019	CS	1,655	48.8	405	96	DD	21
Fiorini et al. (2018)	Italy	2010–2016	2018	CS	1,730	51.1	1424	114	MIC	0.5
Shao et al. (2018)	China	2013–2016	2017	CS	2,283		2283	519	MIC	1
Li et al. (2020)	China	2019–2019	2021	CS	157	10.9	87	48	MIC	0.5
Su et al. (2013)	China	2010–2012	2013	CS	51,891		17731	3810	MIC	1
Hojsak et al. (2012)	Croatia	2001–2010	2012	RET	2,313	12.9	168	20	MIC	1
Hamidi et al. (2020)	Iran	2017–2018	2020	CS	80	50.2	50	11	MIC	0.5
An et al. (2013)	Korea	2009–2012	2013	RET	165		165	20	MIC	1
Shiota et al. (2015)	USA	2009–2013	2015	CS	656		128	6	MIC	1
Li et al. (2017)	China	2009–2015	2017	RET	5,610	14	1746	286	MIC	1
Bolor-Erdene et al. (2017)	Mongolia	2011–2014	2017	CS	320	43.7	152	54	MIC	1
Boehnke et al. (2017)	Peru	2011–2013	2017	CS	109		76	27	MIC	0.5
Ahmad, Zakaria & Mohamed (2011)	Malaysia	2004–2007	2011	CS	777		187	4	MIC	1
Rasheed et al. (2014)	USA	2011–2012	2014	CS	93	47.4	46	22	MIC	1
Guo et al. (2019)	China	2016–2017	2018	CS	346	Range: 1–15	22	8	MIC	1
Jiang et al. (2021)	China	2017–2019	2021	CS	1,533		1533	721	MIC	0.5
Butenko et al. (2017)	Slovenia	2011–2014	2017	RET	107	12	104	25	MIC	8
Tveit et al. (2011)	Alaska	2000–2008	2011	CS	1,181	51	531	159	MIC	1
Tuan et al. (2019)	Vietnam		2019	CS	206	45.3	55	14	MIC	8
Maev et al. (2020)	Russia	2015–2018	2020	CS	27		27	3	MIC	0.5
Figueroa et al. (2012)	Colombia		2012	CS	203	40	146	29	MIC	1
Kim et al. (2011)	Korea	2008–2008	2011	CS	99	54.6	99	26	MIC	1
Adeniyi et al. (2012)	Nigeria		2012	CS	52	Range: 10–90	43	3	DD	30
Yao et al. (2019)	Taiwan	2013–2014	2019	RET	719	61.2	41	14	MIC	1
Honma et al. (2019)	Japan	2012–2015	2018	CS	1,298	14	13	5	MIC	1
Bayati et al. (2019)	Iran	2014–2015	2019	CS	170	Range: 30–75	55	27	MIC	0.5
Pichon et al. (2020)	France	2012–2014	2020	CS	3	33.3	189	1	MIC	0.5
Tanabe et al. (2018)	Japan	2013–2016	2018	RET	1,355		212	50	MIC	1
Karabiber et al. (2014)	Turkey.		2014	CS	159		98	23	DD	30
Saracino et al. (2020)	Italy	2009–2019	2020	NA	3,178	52.3	1646	553	MIC	0.5
Liang et al. (2020)	Taiwan	2013–2019	2020	RET	1,369	54.0 ± 11.9	1369	226	MIC	1
Khademi et al. (2014)	Iran	2011–2012	2014	CS	130		30	4	MIC	1
Milani et al. (2012)	Iran	2010–2011	2012	CS	395	35 ± 19	112	16	MIC	1
Famouri et al. (2018)	Iran	2015–2018	2018	CS	102	8.65 ± 3.88	48	17	MIC	2
Bruce et al. (2019)	Alaska	1998–2006	2019	PRO	362		260	74	MIC	1
Park et al. (2020)	Korea	2017–2019	2020	PRO	174		70	20	MIC	0.5
Binh et al. (2013)	Vietnam	2008–2008	2013	CS	103	44.8	103	34	MIC	1
Keshavarz Azizi Raftar et al. (2015)	Iran		2013	CS	246	45.78 ± 16.23	95	32	MIC	1
Ang et al. (2016)	Singapore	2000–2014	2016	RET	708		708	97	MIC	1
Gościniak et al. (2014)	Poland	2008–2011	2014	CS	165		165	50	MIC	1
Wang et al. (2019)	China	1998–2017	2019	CS	454	50.74 ± 10.942	100	31	MIC	1
Bai et al. (2015)	China	2013–2013	2015	CS	181	44.9	181	56	MIC	0.5
Mégraud et al. (2021)	France	2014–2018	2020	CS	951	52.4 ± 15.7	741	157	MIC	0.5
Sadeghifard et al. (2013)	Iran	2009–2010	2013	CS	50		50	16	DD	20
Bedoya-Gómez et al. (2020)	Colombia		2019	PRO	115	41.8	61	5	MIC	0.5
Miftahussurur et al. (2016)	Japan	2012–2015	2016	PRO	849	49.25	77	7	MIC	0.25
Erkut et al. (2020)	Turkey	2010–2011	2020	PRO	344	39.3	104	29	MIC	1
Zhang et al. (2018)	China	2013	2018	CS	394		136	10	MIC	1
Tsay et al. (2012)	Taiwan	2005–2009	2011	RET	233	55.7	32	2	MIC	1
Mascellino et al. (2018)	Italy	2017	2020	RET	80	59	80	28	MIC	0.5
Khoury et al. (2017)	Israel	2012–2015	2017	RET	107		64	26	MIC	0.5
Saracino et al. (2020)	Italy	2016–2019	2020	RET	270	51.4	221	202	MIC	0.5
Lin et al. (2020)	Taiwan	2008–2017	2019	RET	490	54.5	228	33	MIC	1
Alfizah et al. (2014)	Malaysia	2004–2007	2014	CS	99		161	2	MIC	1
Fasciana et al. (2015)	Italy		2015	CS	100		100	25	MIC	0.5
Ayala et al. (2011)	Mexico	2002–2004	2011	CS/PRO	460		90	9	MIC	2
Picoli et al. (2014)	Brazil	2011–2012	2014	CS	342		54	6	MIC	1
Larsen et al. (2013)	Norway	2008–2009	2012	CS	NA		102	6	MIC	0.5
Kumar et al. (2020)	USA	2009–2019	2019	RET	109		65	39	MIC	0.5
Khademi et al. (2013)	Iran	2011–2012	2013	CS	260	45.8 ± 17.8	78	12	MIC	1
Peretz et al. (2014)	Israel	2011– 2012	2014	CS	176		85	20	MIC	1
Chung et al. (2012)	Korea	2004–2007	2011	CS	185	50.7 ± 14.4	185	20	MIC	1
Ghotaslou et al. (2013)	Iran		2013	CS	123	35 ± 18	123	21	DD	30
Kostamo et al. (2011)	Finland	2000–2008	2010	RET	3,045	62	1037	83	MIC	1
Demiray-Gürbüz et al. (2017)	Turkey	2006–2011	2016	CS	234	43.8 ± 14.0	114	32	MIC	1
Agudo et al. (2011)	USA	2008	2011	CS	118		118	42	MIC	1
Matta, Zambrano & Pazos (2018)	Colombia		2018	CS	409		74	34	MIC	1
Song et al. (2014)	China	2008–2012	2014	PRO/CS	600	42.5 ± 13.2	600	225	MIC	0.5
Wüppenhorst et al. (2014)	Germany	2001–2012	2014	PRO	1,651		1523	475	MIC	1
Shi, Jiang & Zhao (2016)	China		2016	CS	328		328	78	MIC	1
Talebi Bezmin Abadi et al. (2012)	Iran	2009–2010	2011	CS	170	38.6	150	51	MIC	1
Boyanova et al. (2017)	Bulgaria	2011–2016	2017	CS	233	59.1	233	60	MIC	0.5
Manfredi et al. (2015)	Italy	2011–2012	2015	CS	66	9.8	46	12	MIC	4
Morilla et al. (2019)	Spain	2004–2016	2019	RET	3,426	55.7 ± 16.9	1439	278	MIC	0.5
Vekens et al. (2013)	Belgium	2009–2010	2013	PRO	507	48.8	180	24	MIC	1
Maleknejad et al. (2015)	Iran	2012–2014	2015	CS	169	7.30 ± 3.12	21	1	DD	30
Oleastro et al. (2011)	Portugal	2000–2009	2011	PRO	1,115	10.17 ± 4.03	1115	387	MIC	1
Zhang et al. (2015)	China	2009–2010 2013–2014	2015	PRO/CS	1,555	42.4	1321	648	MIC	0.5
Dargiene et al. (2018)	Lithuania	2013–2015	2017	CS	297	32.85	79	2	MIC	0.5
Liu et al. (2011)	China	2009–2010	2011	CS	120	10.0 ± 5.8	73	62	MIC	1
Liu et al. (2018)	China	2010–2016	2017	PRO	1,117		960	247	MIC	1
Tang et al. (2020)	China	2017–2019	2020	CS	400	44.7	117	52	MIC	0.5
Bachir et al. (2018)	Algeria	2012–2015	2017	CS	200		151	38	MIC	0.5
Seck et al. (2013)	Senegal	2007–2009	2013	CS	108	45.3	108	1	MIC	1
Karczewska et al. (2011)	Poland	2006–2008	2011	CS	115		115	39	MIC	1
Lee et al. (2019)	South Korea	2003–2018	2019	PRO	740	56.3	740	280	MIC	1
Raaf et al. (2017)	Algeria	2015–2016	2017	PRO	147		43	16	DD	17
Hansomburana et al. (2012)	Thailand	2006–2008	2012	PRO	200	52.8	82	11	MIC	1
Mirzaei et al. (2013)	Iran	2011–2011	2013	CS	110	34	48	7	MIC	1
Lee et al. (2013)	Korea	2003–2012	2013	PRO	433	55.53	433	127	MIC	1
Shokrzadeh et al. (2015)	Iran	2010–2011	2014	CS	197	46	111	29	MIC	1
Oporto et al. (2019)	Chile	2018	2019	CS	229	50.68	44	18	MIC	0.5
Aumpan et al. (2020)	Thailand	2019	2020	CS	58	43.8	14	4	MIC	0.5
Vilaichone et al. (2020)	Thailand	2010–2015	2020	CS	1,178	41.5	357	7	MIC	0.5
Cerqueira et al. (2011)	Portugal		2011	CS	NA		33	21	MIC	1
Binyamin et al. (2017)	Israel	2015–2016	2017	CS	85		54	34	MIC	1
Camorlinga-Ponce et al. (2021)	Chile	1997–2017	2021	CS	167	50.72	167	15	MIC	0.5
Biernat et al. (2020)	Poland	2016–2019	2020	RET	108	12.5	91	28	MIC	0.5
Trespalacios et al. (2013)	Colombia	2009–2011	2013	CS	256		276	42	MIC	1
Lok et al. (2020)	China	2018–2019	2020	CS	176	48.4.	65	34	MIC	0.5
Bahmaninejad et al. (2021)	Iran	2020–2020	2021	CS	100		50	33	MIC	1
Draeger et al. (2015)	Germany	2004–2013	2015	RET	481		481	409	MIC	1
Zerbetto De Palma et al. (2017)	Argentina	2011–2013	2015	CS	52		52	14	MIC	0.5
Boyanova et al. (2012)	Bulgaria	2004–2010	2012	CS	519	52.16	519	93	MIC	1
Tshibangu-Kabamba et al. (2020)	Congo	2017–2018	2020	CS	220	45.3 ± 15.3	102	24	MIC	0.5
Okuda et al. (2017)	Japan	1997–2013	2016	RET	332	11.6 ± 3.4	76	33	MIC	1
Vilaichone et al. (2013)	Thailand	2004–2012	2013	CS	3,964	53.3	400	15	MIC	0.5
Zhang et al. (2020)	China	2017–2019	2020	CS	238		238	84	MIC	0.5
Zhang et al. (2020)	China	2012–2014	2020	CS	79	9.7 ± 2.8	79	29	MIC	1
Mansour et al. (2016)	France	2009–2009	2015	PRO	149	53.65	42	12	MIC	1
Kuo et al. (2021)	Taiwan	2017–2020	2021	CS	64	53.8	41	38	MIC	0.5
Miendje Deyi et al. (2011)	Belgium	1990–2009	2011	CS	9,430	29.3	9430	524	MIC	1
Han et al. (2016)	China	2015–2015	2016	CS	325	47.2	325	65	MIC	1
Bińkowska et al. (2018)	Italy	2008–2016	2018	CS	170		170	29	MIC	1
Bachir et al. (2018)	Algeria	2014–2016	2018	PRO	270		212	53	MIC	0.5
Hanafiah et al. (2019)	Malaysia	2014–2015	2019	CS	288	52.41 ± 16.44	59	21	MIC	1
Vazirzadeh et al. (2020)	Iran	2018–2018	2020	CS	165	50:3 ± 15:5	83	21	MIC	0.5
Rezaei, Abadi & Mobarez (2020)	Iran	2015–2018	2019	CS	200	54	73	17	MIC	0.5
Yakoob et al. (2013)	Pakistan	2008– 2010	2013	CS	120	41 ± 13	47	17	MIC	1
Gehlot et al. (2016)	India	2011–2013	2016	CS	483	43	68	8	MIC	0.5
Boyanova et al. (2013)	Bulgaria	2007– 2012	2013	RET	588		588	118	MIC	1
Boyanova et al. (2015)	Bulgaria	2012–2014	2015	CS	53	50.7	53	9	MIC	0.5
Otth et al. (2011)	Chile		2010	CS	240	54.5 ± 15.7	88	8	MIC	2
McNulty et al. (2012)	Uk	2009–2010	2012	CS	2,063		241	86	MIC	1
Wang et al. (2018)	China	2013–2014	2018	CS	NA		100	13	MIC	0.5
Alavifard et al. (2021)	Iran	2017–2019	2020	CS	82	49.7 ± 3.33	82	36	MIC	0.5
Regnath et al. (2017)	Germany	2002–2015	2016	RET	582	12 years	608	75	MIC	0.5
Lu et al. (2019)	Taiwan	1998–2018	2019	RET	70	13.2 ± 3.2	70	16	MIC	1
Di Giulio et al. (2016)	Italy	2010–2014	2015	CS	115		181	131	MIC	0.5
Enany & Abdalla (2015)	Egypt		2015	CS	150		107	6	DD	40
Trespalacios et al. (2015)	Colombia		2014	CS	127		107	42	MIC	1
Gatta et al. (2018)	Italy	2010–2015	2018	RET	1,682		1325	478	MIC	0.5
Goudarzi et al. (2016)	Iran	2015–2015	2016	CS	154		110	28	MIC	1
Bayati et al. (2020)	Iran		2019	CS	170	30 ± 75.	55	27	MIC	0.5
Dang et al. (2020)	Vietnam	2014–2016	2020	CS	153	38.3 ± 10.7	153	111	MIC	1
Phan et al. (2015)	Vietnam	2012–2014	2014	CS	92	44.1 ± 13.4	92	39	MIC	1
Khashei et al. (2016)	Iran	2014–2014	2016	CS	318	41.5	100	20	MIC	1
Shetty et al. (2019)	Australia	2014–2017	2019	CS	180	46.2 ± 14	113	23	MIC	0.5
Macías-García et al. (2017)	Spain	2014–2016	2017	PROCS	217	64	76	17	MIC	1
Farzi et al. (2019)	Iran	2016–2017	2019	CS	160	46.5 ± 8.3	68	23	MIC	1
Lyu et al. (2020)	China	2016–2018	2020	PRO	1,113	43	791	271	MIC	0.5
Shmuely et al. (2020)	Israel	2013–2017	2020	RET/CS	128	45	128	70	MIC	256
Ogata et al. (2013)	Brazil	2008–2009	2013	CS	77	11.1 ± 3.9	77	15	MIC	2
Abadi et al. (2011)	Iran	2008–2010	2011	CS	147	34.5	147	32	MIC	1
korn Vilaichone et al. (2017)	Thailand	2016–2016	2017	CS	148	56.3 ± 13.3	50	1	MIC	0.5
Ferenc et al. (2017)	Poland	2011 and 2013	2016	CS	185	49 ± 16.8	67	37	MIC	1
Azzaya et al. (2020)	Mongolia	2014–2016	2020	CS	361	44.3 ± 13.4	361	108	MIC	0.5
Mi et al. (2021)	China	2018–2018	2021	CS	48		65	21	MIC	0.5
Boyanova et al. (2014)	Bulgaria	2012–2013	2014	CS	50	50.5	50	11	MIC	0.5
Boyanova et al. (2016)	Bulgaria	2010–2015	2015	CS	299	47.3	299	84	MIC	0.5
Megraud et al. (2021)	France	2018–2019	2021	PRO	1,211	51.2	1211	259	MIC	0.5
Megraud et al. (2013)	France	2008–2009	2013	PRO	2,204		2204	431	MIC	1
Ducournau et al. (2016)	France	2014–2015	2016	CS	984	51.5 ± 15.9	266	59	MIC	1
Bouihat et al. (2017)	France	2015–2016	2016	PRO	255	47.5	177	45	MIC	0.5
Fernández-Reyes et al. (2019)	Spain	2014–2017	2019	PRO	112		99	12	MIC	0.5
Saniee et al. (2018)	Iran	2010–2017	2018	CS	985		218	75	DD	2
Mokhtar et al. (2019)	Malaysia	2015–2016	2019	CS	352	52	13	4	MIC	0.5
Montes et al. (2015)	Spain	2008–2012	2014	RET	143		74	25	MIC	1
Deyi et al. (2019)	Belgium	2015–2016	2019	CS	846		846	141	MIC	0.5
Tang et al. (2020)	China	2016–2019	2020	CS	NA		301	201	MIC	0.5

### Study selection

All records obtained from online databases were imported into EndNote (Version 20), and duplicates were eliminated. M-H and S-K independently assessed the titles and abstracts; V-H-K resolved discrepancies. Studies were considered to be appropriate for the analysis if they presented data concerning the prevalence of *H. pylori* resistant to CLA. An English language restriction was imposed, while abstracts, conferences, case reports, case series, reviews, studies with unclear results, and duplicate articles were excluded from the analysis.

### Data extraction

Our study included studies based on pre-defined criteria and evaluated as full-text articles. Two reviewers conducted the data extraction process independently (M-H, S-K). Any discrepancies were discussed and resolved by consensus of the two reviewers. The primary outcome of focus was the prevalence of clarithromycin-resistant *Helicobacter pylori*. Information extracted from each study included the first author’s name, year of publication, geographical location, antimicrobial susceptibility testing method, breakpoints for interpretation of the test results, sample size, and the number of clarithromycin-resistant *H. pylori*. All extracted data are available in an accompanying Supplemental File.

### Quality assessment

Two reviewers (S-K and M-H) evaluated the quality of the studies using the Newcastle Ottawa Scale (NOS). In cases of disagreement, a third author (M-SH) was consulted to determine a consensus. The assessment of the studies was based on three criteria: selection, comparability, and exposure/outcome assessments.

### Statistical analysis

For the present study, the sample size of isolates for antimicrobial susceptibility testing (AST) and the number of resistances to each antibiotic were used to calculate a weighted pooled resistance and their 95% confidence intervals. In order to prevent the exclusion of studies from the meta-analysis due to 0 or 100 resistance prevalence, the Inverse of Freeman-Tukey double arcsine transformation was conducted using Metaprop command in STATA software (version 17.1). A random-effects model was implemented to estimate pool proportions (Egger et al., 1997; Harbord et al., 2010). The I^2^ with a *P* ≤ 0.05 was used to identify significant heterogeneity. The presence of a small-study effect or publication bias was assessed using Egger’s linear regression test and Begg’s test (Harbord, Harris & Sterne, 2009). Subgroup analyses were conducted to determine the impact of the country, continent, publication year (2010–2017, 2018–2021), (AST) (Disc diffusion, Gradient methods), and breakpoints for interpretation of AST results on the variation.

## Results

### Descriptive statistics

**Figure 1:**
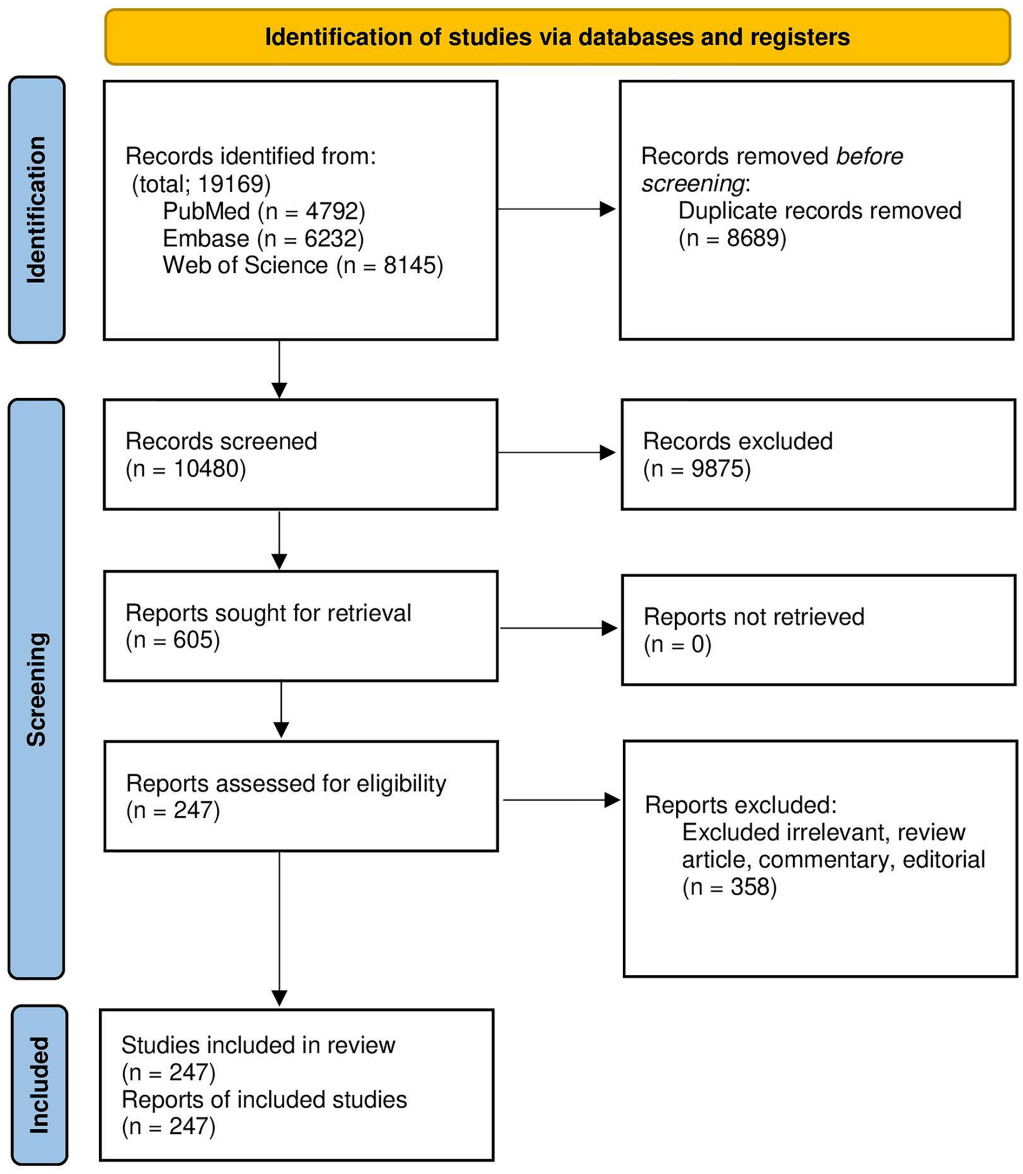
The study PRISMA flow diagram.

In this research, 19,169 records were acquired in EndNote version 20, a reference manager software. A total of 8,689 duplicated articles were then removed, leaving a total of 247 eligible studies that were included in the systematic review and meta-analysis. The screening and selection presage were summarized in the PRISMA flow chart (Fig. 1). Overly 20,936 *H. pylori* isolates have been investigated in included articles. More than half of the isolates were investigated in Asia (55.10% Isolated). Although most pieces were from Iran (38 articles), the highest number of isolates among the countries was that investigated from China (32,130 Isolates, 36.52% of total isolates). Description data are summarized in Table 2.

**Table 2:** Clarithromycin-resistant *Helicobacter pylori* prevalence. 95% Confidence Intervals (CI) were used. *P* ≤ 0.05 was considered statistically significant.

	No of article	Clar-resistant, Total isolates	Proportion (LCI, HCI)	Weight	I^2^ (*P*)
Overall	248	8736, 87991	27.53 (25.41, 29.69)	100.00	97.80% (*P* = 0.00)
2010–2017	143	12891, 60452	24.28 (21.7, 26.96)	57.68	97.91% (*P* = 0.00)
2018–2021	105	8045, 27476	32.14 (28.69, 35.69)	42.32	97.24% (*P* = 0.00)
Iran	38	1193, 3628	27.24 (21.68, 33.18)	14.91	93.14% (*P* = 0.00)
Finland	1	83, 1037	8.00 (6.43, 9.83)	0.43	NA
Chile	4	137, 704	18.56 (8.47, 31.34)	1.62	91.76% (*P* = 0.00)
Brazil	4	40, 247	15.29 (9.79, 21.7)	1.55	38.94% (*P* = 0.18)
Romania	1	22, 70	31.43 (20.85, 43.63)	0.40	NA
Austria	1	16, 96	16.67 (9.84, 25.65)	0.41	NA
France	8	990, 4873	21.13 (15.26, 27.66)	3.31	95.23% (*P* = 0.00)
Eastern Cape	1	40, 200	20 (14.69, 26.22)	0.42	NA
Spain	8	1161, 4650	27.41 (17.03, 39.18)	3.30	98.22% (*P* = 0.00)
Malaysia	5	38, 522	10.2 (1.59, 23.94)	1.91	93.33% (*P* = 0.00)
Alaska	2	233, 791	29.45 (26.31, 32.68)	0.86	NA
Korea	5	213, 952	20.59 (12.26, 30.37)	2.07	90.69% (*P* = 0.00)
Taiwan	10	453, 2088	29.16 (15.9, 44.45)	3.92	96.85% (*P* = 0.00)
Mexico	2	17, 135	12.3 (7.14, 18.53)	0.78	NA
USA	5	347, 1157	32.98 (17.21, 50.95)	2.03	95.84% (*P* = 0.00)
Portugal	5	541, 1428	48.11 (30.07, 66.41)	1.97	95.52% (*P* = 0.00)
China	32	8227, 32130	34.05 (29.33, 38.92)	13.14	98.16% (*P* = 0.00)
Poland	6	178, 601	29.77 (18.41, 42.52)	2.42	90.49% (*P* = 0.00)
Belgium	3	689, 10456	11.28 (3.95, 21.67)	1.29	NA
Turkey	7	170, 684	25.78 (19.44, 32.67)	3.22	76.74% (*P* = 0.00)
Croatia	1	20, 168	11.9 (7.43, 17.79)	0.42	#VALUE!
Colombia	5	152, 664	24.26 (12.96, 37.68)	2.04	92.33% (*P* = 0.00)
Nigeria	2	44, 147	28.22 (21.13, 35.86)	0.78	NA
Norway	1	6, 102	5.88 (2.19, 12.36)	0.41	NA
Thailand	7	54, 1097	6.24 (2.73, 10.86)	2.73	81.45% (*P* = 0.00)
Bulgaria	6	375, 1742	21.89 (18.2, 25.81)	2.48	66.49% (*P* = 0.01)
UK	1	86, 241	35.68 (29.64, 42.09)	0.42	NA
South Korea	7	560, 1778	31.4 (19.68, 44.43)	2.88	96.35% (*P* = 0.00)
Germany	4	971, 2771	32.08 (6.55, 65.66)	1.71	99.64% (*P* = 0.00)
Vietnam	5	254, 507	45.72 (28.85, 63.11)	2.02	93.56% (*P* = 0.00)
Senegal	1	1, 108	0.93 (0.02, 5.05)	0.41	NA
Pakistan	2	22, 139	13.33 (8.04, 19.63)	0.78	NA
Australia	2	31, 124	23.47 (16.01, 31.75)	0.67	NA
Japan	12	854, 2494	35.89 (27.02, 45.26)	4.68	93.72% (*P* = 0.00)
India	3	63, 216	25.25 (2.81, 59.01)	1.19	NA
Italy	11	1663, 5367	40.38 (25.65, 56.04)	4.55	99.12% (*P* = 0.00)
Israel	6	272, 597	46.12 (35.66, 56.75)	2.39	84.00% (*P* = 0.00)
Bangladesh	1	22, 56	39.29 (26.5, 53.25)	0.39	NA
Canada	1	8, 20	40.00 (19.12, 63.95)	0.32	NA
Argentina	1	14, 52	26.92 (15.57, 41.02)	0.38	NA
Egypt	2	18, 127	10.61 (5.53, 16.89)	0.73	NA
Singapore	1	97, 708	13.70 (11.25, 16.46)	0.43	NA
Dominican	1	2, 64	3.13 (0.38, 10.84)	0.39	NA
Iceland	1	9, 105	8.57 (3.99, 15.65)	0.41	NA
Mongolia	2	162, 513	31.54 (27.57, 35.64)	0.84	NA
Peru	1	27, 76	35.53 (24.88, 47.34)	0.40	NA
Slovenia	1	25, 104	24.04 (16.2, 33.41)	0.41	NA
Lithuania	1	2, 79	2.53 (0.31, 8.85)	0.40	NA
Algeria	3	107, 406	26.62 (21.42, 32.15)	1.21	NA
Russia	3	28, 333	13.34 (2.11, 30.9)	1.12	NA
Honduras	1	13, 116	11.21 (6.1, 18.4)	0.41	NA
Switzerland	2	145, 216	67.16 (60.71, 73.31)	0.81	NA
Cameroon	1	19, 140	13.57 (8.37, 20.38)	0.41	NA
Congo	1	24, 102	23.53 (15.69, 32.96)	0.41	NA

**Note:**

High confidence interval, HCI; low confidence interval, LCI; I-squared, I^2^; Degrees of freedom, DF.

### Publication bias

**Figure 2:**
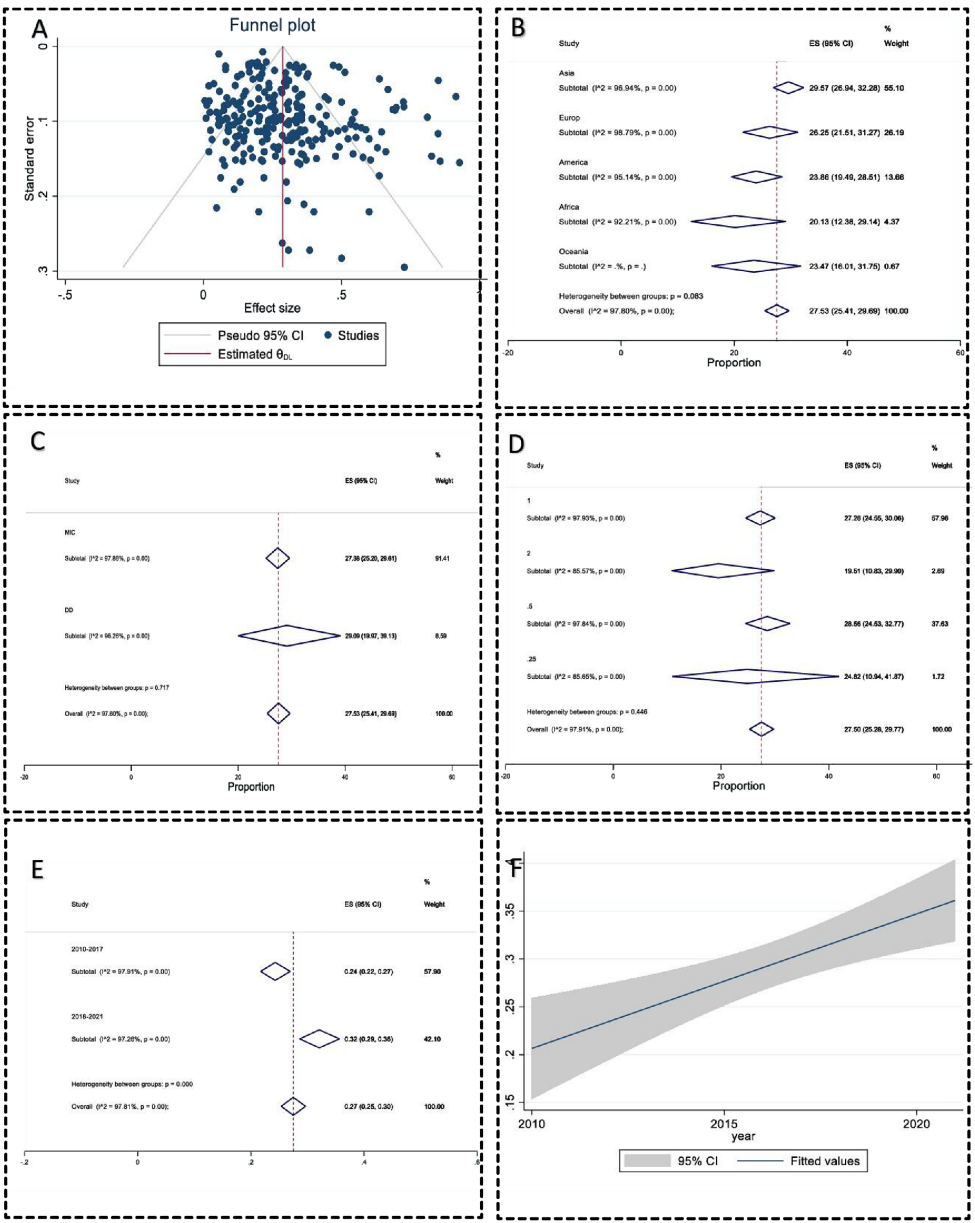
Meta-analysis charts. (A) The funnel plot of clarithromycin-resistant *Helicobacter pylori* prevalence did not have significant evidence of publication bias; (B) the subgroup analysis forest plot of clarithromycin-resistant *Helicobacter pylori* prevalence in different continents; (C) the subgroup analysis forest plot of clarithromycin-resistant *Helicobacter pylori* prevalence using different AST methods; (D) subgroup analysis forest plot of clarithromycin-resistant *Helicobacter pylori* prevalence in different breakpoints to interpret antimicrobial susceptibility test data; (E) subgroup analysis forest plot of clarithromycin-resistant *Helicobacter pylori* prevalence in years; (F) the regression analysis of clarithromycin-resistant *Helicobacter pylori* prevalence over years with 95% Confidence interval had a significant correlation 0.013 (95% CI [0.01–0.02]) (*P* < 0.001).

The publication bias was significant by the regression-based Egger test for small-study effects (*P* = 0.04), but Begg’s test for small-study effects was insignificant (*P* = 0.09). The Nonparametric trim-and-fill analysis of publication bias also did not change the effect size. The funnel plot also did not have significant evidence of publication bias (Fig. 2A). The sensitivity analysis or one leave-out method also had no significant bias.

### Meta-analysis

In 248 included studies, 20,936 isolates have been investigated, and 8,736 isolates have been reported as resistant. The pooled prevalence of CLA-resistance *H. pylori* was 27.53 (95% CI [25.41–29.6]). Heterogeneity between reports was significant (I^2^ = 97.80, *P* < 0.01). The heterogeneity between countries was substantial (*P* < 0.001). Switzerland, Portugal, and Israel had the highest resistance rates (67.16%, 48.11%, and 46.12%, respectively), and Senegal, Lithuania, and the Dominican Republic had the lowest resistance prevalence, 0.93%, 2.53%, and 3.13%, respectively) (Table 2). The heterogeneity between the continent subgroups was insignificant (*P* > 0.05) (Fig. 2B). The heterogeneity between the AST methods subgroup was insignificant (Fig. 2C). The breakpoints for the interpretation AST subgroup were insignificant (*P* > 0.05) (Fig. 2D). The CLA-resistant *H. pylori* prevalence increased from 24.28% in 2010–2017 to 32.14% in the 2018–2021 years period (*P* < 0.01) (Fig. 2E). All statistics are summarized in Table 2. The regression meta-analysis for resistance rate over the publication year had a significant correlation of 0.013 (95% CI [0.01–0.02]) (*P* < 0.001) (Fig. 2F).

## Discussion

Over the past years, the treatment of *H. pylori* infections has been performed using the standard triple therapy regimen, including CLA, a proton pump inhibitor, with either metronidazole or amoxicillin (Gong et al., 2020). However, in recent years, it is revealed that some *H. pylori* isolates have developed resistance to CLA (Sanches et al., 2016). Therefore, the efficacy of the standard triple therapy regimen is in decline. In 2017, WHO listed the CLA-resistant *H. pylori* among antibiotic-resistant priority pathogens that need research and development of new antibiotics (Khani, Abadi & Mobarez, 2019). Globally, surveillance and being aware of the frequency of resistance to antibiotics among pathogens is critical, and obtained results can be helpful in different sections such as the design of screening or follow-up programs, and the development of antimicrobial stewardship programs (Azimi et al., 2019; Pormohammad, Nasiri & Azimi, 2019).

In the present systematic review and meta-analysis study, we surveyed and analyzed the worldwide prevalence of CLA resistance among *H. pylori* isolates from 2010 to 2021. The awareness of CLA resistance among different countries of the world and effective treatment of *H. pylori* infections are the main goal of the current study. The present systematic review and meta-analysis study included 247 eligible studies from 54 different countries. Our analyses revealed that the overall prevalence of clarithromycin-resistance *H. pylori* was 27.53%, worldwide.

Resistance to CLA among *H. pylori* is occur in two different levels including (1) a high level of resistance (MIC more than 64 mg l^−1^) and (2) a low level of resistance (0.5 ≤ MIC ≤ 1 mg l^−1^) (He et al., 2021). Point mutations, multidrug efflux pump systems, and synergistic effect of mutations in genes *rpl22 (*ribosomal protein L22) and *infB* (translation initiation factor IF-2) with 23S rRNA point mutations are the main CLA resistance mechanisms among *H. pylori* isolates (Marques et al., 2020; Li et al., 2021). Moreover, it is presumed that some outer-membrane proteins have a role in CLA resistance in *H. pylori* isolates (Marques et al., 2020). In the Western world and among developed countries, more than 90% of CLA resistance is related to point mutations in the peptidyl transferase region of the V domain of 23S rRNA gene (Mégraud, 2004). The main point mutations related to CLA resistance are A2142G, A2143G (adenine-to-guanine transition at either position 2142 or 2143), A2142C (adenine-to-cytosine transversion at position 2142), A2115G, A2144T, G2141A, G2144T, T2289C, T2717C, and C2694A (Gong et al., 2020; Marques et al., 2020; Li et al., 2021). Moreover, *hp1181* and *hp1184* mutations are associated with CLA resistance (Li et al., 2021). Mutation in the 2142 and 2143 positions leads to restricted resistance and different levels of resistance, respectively (Kim et al., 2020).

In the present research, more than half of the included studies were performed in Asia. These results demonstrated that CLA resistance is a main public health issue in most Asian countries. Among studies surveyed CLA resistance rates in 54 different countries, Switzerland (67.16%) and Senegal (0.93%) had the highest and lowest resistance rates, respectively. The high level of CLA resistance can be due to the following reasons: (1) inappropriate prescription and unregulated or widespread use of CLA, and (2) the use of CLA in other infections such as respiratory tract infections or intestinal parasites infections (Chen et al., 2017). Time trend analyses revealed that the CLA-resistant rates among *H. pylori* isolate increased from 24.28% in 2010–2017 to 32.14% in the 2018–2021 years’ period. An increase in CLA resistance rates is an alarming finding. In areas where CLA-resistance is more than 15%, it is recommended to perform susceptibility testing before prescribing the standard triple therapy regimen (Sanches et al., 2016; Abadi, 2017). Combination therapy with other drugs such as tinidazole can be helpful in the treatment of *H. pylori* infections. It is revealed that CLA combined with tinidazole can reduce the CLA resistance rate, decrease inflammatory reactions, and can effectively eliminate *H. pylori* infections (He et al., 2021). One of the limitations of this study was that we evaluated the CLA resistance rate only and the other antibiotics were not considered.

## Conclusion

Our analysis revealed that CLA resistance rates varied among studies performed in different 54 countries. Altogether, results showed that the overall CLA resistance rate is 27.53%, worldwide. The difference in CLA resistance rate among the included studies can be due to several reasons such as differences in antibiotic prescription rates in various geographic areas, use of different MIC breakpoints or inaccurate criteria in performed studies, and the emergence of multidrug-resistant (MDR) strains. We performed a time trend analysis and the results revealed that the clarithromycin-resistance rates in increasing in recent years. Based on our findings, systematic surveillance, and proper monitoring of CLA resistance rates, as well as monitoring the use of CLA in patients, and performing the CLA susceptibility test before prescription may be critical actions for the inhibition and control of *H. pylori* infections.

## Supplemental Information

10.7717/peerj.15121/supp-1 Supplemental Information 1 Prisma 2009 checklist.

10.7717/peerj.15121/supp-2 Supplemental Information 2 Characteristics of studies included in the meta-analysis.

## Additional Information and Declarations

### Data Availability

The following information was supplied regarding data availability: The raw measurements are available in the Supplemental File.
